# A Cross-Sectional Study about Knowledge, Awareness and Perception of Risk Factors for Cancer among Cancer-Patients Relatives and Healthy Adults in Ho Chi Minh City, Vietnam

**DOI:** 10.31557/APJCP.2021.22.1.277

**Published:** 2021-01

**Authors:** Dung X Pham, Tien T T Pham, Thang N Pham, Tung D Bui

**Affiliations:** 1 *Ho Chi Minh City Oncology Hospital, Vietnam. *; 2 *Pham Ngoc Thach University of Medicine, Ho Chi Minh City, Vietnam. *; 3 *Department of Psychology, Hoa sen University, Ho Chi Minh City, Vietnam. *

**Keywords:** Ho Chi Minh city, cancer burden, cancer KAB

## Abstract

**Background and Aims:**

Although cancer is common in Ho Chi Minh city, Vietnam, the community awareness is still unknown. The primary objective of this study was to examine and compare the knowledge and risk perceptions of cancer possessed by cancer patients - relatives and healthy adults in Ho Chi Minh City, Vietnam.

**Methods:**

A cross-sectional study was conducted from June to August 2019. Cancer patients and their relatives were drawn from those who were hospitalized in the Oncology Hospital, Ho Chi Minh City. Healthy individuals were those without a known diagnosis of cancer, and they were drawn from the participants of the Vietnam Osteoporosis Study. A total of 533 participants including 249 patients and relatives (cancerous group) and 284 healthy individuals (healthy group), were asked to respond to a structured questionnaire that was comprised of items concerning cancer knowledge, risk factor perception, and general attitude towards cancer, using Yes, No, or Likert Scale for response.

**Results:**

The findings showed that patients hold poorer knowledge of pathology, signs, symptoms, prevention, and treatment and lower awareness of risk factors but more positive attitude towards cancer as compared to their healthy counterparts. Overall, both groups varied in their cancer knowledge, with many areas remain to be improved.

**Conclusions:**

Knowledge about cancer and its risk factors should be improved among the general population as well as among those with direct experiences with cancer. Practical implications: The findings provided by this study has major implications for the design of an educational program for cancer patients in clinical settings and awareness programs for the general public as a primary preventive measure for mitigating the cancer burden. Future studies with larger and more diverse samples or qualitative studies exploring the personal narratives of people living with cancer could take advantage from the preliminary data provided by this study.

## Introduction

Cancer is a serious health problem worldwide. In 2018, there was an estimated 18.1 million new cases of cancer and 9.6 million deaths from the disease; by 2040 thepredicted global burden will double to about 29-37 million new cancer caseswith the greatest increases in low and middle income countries (WHO, 2020). A response to the increase cancer burden requires the accurate understanding of the disease and the potential impact of programmes and policies. Cancer are cause by mutations that may be inherited, induced by environmental factors or results from DNA replication error (Tomasetti et al., 2017). It does mean that most of cancers can be prevented by primary prevention by changes in the environment; moreover, secondary prevention with early detection and intervention are also important to reduce deaths from the many cancers arising from unavoidable replication error mutations.

There is the discrepancy between consensus statements from the cancer literacy with the perceptions of cancer among the general public (Richards et al., 2017). Provision of accurate and accessible information with concerns about cancer information overload and the resulting disempowering public perceptions with a lots of dispelling myths and misconceptions. Furthermore, poor awareness of cancer etiology, risk factors resulting in delayed presentation as well as low availability of screening programs and limited access to health care services contribute to cancer related deaths. 

Because most of the cancer risk factors are modifiable, it is expected that a large proportion of cancers are preventable. Indeed, studies from the US (Marlow et al., 2012) and UK (Tomasetti et al., 2017) suggest that about 40% of all incident cancer cases (excluding skin cancer) and 45% of cancer deaths were preventable. In the analysis (Marlow et al., 2012), cigarette smoking was identified as the most important risk factor with the attributable fraction being 19% of cancers and ~29% of cancer deaths. Thus, the modification of lifestyle could be an effective preventive measure of cancer in the general community. 

Modification of risk factors, including life factors, requires the awareness and knowledge of etiology and risk factors (Diviani and Schulz, 2011). There is evidence that when individuals are aware of risk factors, they tend to be more active in the participation in cancer control programs (Diviani et al., 2014). However, the awareness as well as knowledge of cancer risk factors differ among individuals, depending on their personal experiences, education, life narrative, and perhaps relationships (Lipworth et al., 2010). The difference in the awareness and knowledge of cancer risk was also observed among ethnicities (Marlow et al., 2012), suggesting a link to cultural factors. 

Vietnam is a lower middle-income country that has been undergone a rapid socio-economic transformation over the two decades. The socio-economic changes have happened concomitantly with changes in lifestyle and newly introduced risk factors related to non-communicable diseases (NCDs) including specific types of cancer and diabetes, such as environmental pollution, cigarette smoking, excessive alcohol intakes, and sedentary life style. Besides, increased life expectancy and aging population are also contributing to new cancer cases in Vietnam (Vuong et al., 2010). Although there has been no comprehensive cancer prevalence study in Vietnam, the International Agency for Research on Cancer (IARC) statistics showed that in 2018 alone, there were ~165,000 (or 0.17% of the population) new cancer cases diagnosed, and ~115,000 deaths attributable to cancer (IARC, 2020). However, in contrast to more economically developed countries where cancer prevalence, its risk factors, and people’s awareness of cancer have been well documented, there a few such data in Vietnam. Therefore, the aim of this study is to evaluate the awareness of cancer risk, the extent of knowledge, and the perception of risk factors among the general community, cancer patients and their relatives in Vietnam. 

## Materials and Methods

*Study Design and Methods*

*Participants*

This cross-sectional study had been conducted from June to August, 2019 in Ho Chi Minh City, Vietnam. Participants (n = 533) were divided into two group: cancer patients, their relatives, and ‘healthy people’ from the general community. Cancer patients and their relatives were drawn from those who were hospitalized in the Oncology Hospital, Ho Chi Minh City. Healthy individuals were those without a known diagnosis of cancer, and they were drawn from the participants of the Vietnam Osteoporosis Study (Ho-Pham and Nguyen, 2017). The study’s protocol and procedure were approved by the Ethics Committee of Oncology Hospital, Ho Chi Minh City, and individuals gave written informed consent. 

Inclusion criteria: Those who are in the cancerous group and the healthy group and willing to response to the questionnaires are included in the study. 

*Exclusion criteria*

- Mentally ill 

- Unable to hear, read or speak

*Questionnaire*

We developed a structured questionnaire that draws on knowledge about cancer from published research worldwide (Trieu et al., 2015; Vu and Bui, 2012; Wardle et al., 2001). The questionnaire was first developed in English and then translated into Vietnamese. The questionnaire was divided into four main sections: (i) demographic and bio-data concerning a participant; (ii) general knowledge about cancer (29 questions); (iii) risk factors for cancer (31 questions); and (iv) attitude toward the diagnosis and treatment of cancer (16 questions). The full list of questions is available in the appendix. We conducted a test-retest reliability study of thequestionnaire in a sample of 30 participants in Ho Chi Minh City, with anintervening period of two weeks. The coefficient ofreliability ranged between 0.75 and 0.94 for the 76questions. There was no significant difference in thereliability coefficients between men and women. Analysisof reliability by age group was not conducted because ofthe limited sample size.

For the knowledge part, there are 21 items, and each had two possible answers (Yes/No). For example, the first question asks whether “most cancers are caused by genetics / hereditary”, the correct answer would be “No” which was coded as “1”. In another 8 items which are structured as multiple-choice questions with one correct answer per question. For the second part (e.g. risk factors questions), there were 4 possible answers: decrease the risk of cancer; increase the risk; no evidence; and don’t know. Each correct answer was given one point and incorrect answers were given no point. The last part of the questionnaire includes 16 items concerning information about cancer and 8 items on ‘feeling’. Each of those items had 5 answers according to the Likert scale: absolutely correct; correct; no opinion; incorrect; and absolutely incorrect. Every correct answer was given one point, while other answers had no point.

The questionnaire was administered to each participant for about 1 hour. Participants filled in the questionnaire, and if they were not clear, they could ask a research team memberfor explanation. The questionnaire was collected bya research team member within the same day. All respondents received no financial bonus for filling out the questionnaire, but they received free health check-up from doctors. 

*Analysis*

Data were checked and stored in a computer database. The analysis of data was mostly descriptive using the “likert” package within the R Statistical Environment (RDCT, 2014). We treated the sum of scores for each scale (e.g. knowledge, risk, information, and feeling) as a continuous variable (Sullivan and Artino, 2013). Test of hypothesis of difference between groups (e.g. patients, and community) was conducted by the Chi-squared statistics for categorical variables, and analysis of variance for continuous or continuous-like variables. A P-value of less than 0.05 was considered statistically significant. 

## Results

*Characteristics of participants*

The demographic characteristics of the study participants are presented in Table 1. The study involved 533 individuals (N = 533), including 249 cancer patients and relatives (cancerous group, mean age: 42.7, SD: 15), and 284 healthy adults who are not known to have cancer (healthy group, mean age: 34.6, SD: 13.4). Approximately 66% of the participants were women (n = 350). About one-third of participants had university education, 34.2% were on full-time employment, and 70% earnt less than 871 USD per month. There were significant differences between the cancerous and healthy groups for age, gender, educational level, income, employment status, and marital status (Table 1). In general, the cancerous group were older and had more females, lower education, lower income, more homework and retired than healthy group.

*Knowledge of cancer*

Overall, there were a significant difference between cancerous (35.01 ± 8.41) and healthy groups (37.78 ± 8.06) for mean total knowledge (Table 2), but the discrepancy did not exist after being adjusted for education level.Cancer patients and relatives were less awarecompared with their healthy counterparts about risk factors; but more positive in general attitude towards cancer than healthy population. Such differences remain significant after being adjusted for education level. There was no significant difference in awareness of cancer etiology, signs and symptoms, and prevention and treatment between two groups. 

Among healthy individuals, except for educational level, no otherdemographic characteristics including age, gender, income level, employment status, or marital status predicted total knowledge score. While in patients and their relatives, except age and gender, other factors includingeducational level, income, employment status, and marital status were significantly associated with total knowledge score(Table 3). The vast majority ofparticipants were aware that cancer is characterised by uncontrolled cell growth (90.6%) and that cancer is a non-contagious disease (89.1%). In contrast, very few people knew that cancer has genetic origin, with only 34.5% of cancer patients and relatives and 38.7% of healthy people agreeing with the statement ‘Most cancer is due to genetic’ (Table 4). 

Signs, symptoms, and diagnosis. While most participants knew about stage of cancer (93.8%), pathology biopsy (77.5%), and breast cancer incidence in women (82.4%); only over half of participants were aware that cancer might not always be associated with pain (55.9%) and that cancer can be diagnosed evenwhen symptoms are subtle(50.7%) (Table 4). Similarly, few participants understood about pap smear use for cervical cancer screening (65.3%) as well as about faecal occult blood test (FOBT) for colorectal cancer screening (36.2%). In general, healthy individuals demonstrated higher awareness of all signs and symptoms of cancer than cancer patients and their relatives except for the likelihood of breast cancer incidence in men (65.8% vs. 68.3%). Much fewer cancer patients and relatives than healthy adults had good understanding of cancer pain (49% vs. 62% respectively, p = .003), early diagnosis possibility (43% vs. 57.4%, p =.001), pap smear for cervical cancer screening (59.8% vs. 70.1%, p = .014), and FOBT for colorectal cancer screening (24.5% vs. 46.5%, p < .001). 

Prevention and treatment. Most participants displayed general knowledge of cancer prevention and treatment with 84.7% of patients and relatives and 88% of healthy adults were aware that cancer is preventable and 83.5% of all participants thought that cancer is curable. However, few participants (32.1%) perceived that cancer treatment might not only involve radiotherapy or chemotherapy; and less than half of participants (46.9%) understood about palliative care (Table 4). Noticeably, patients and relatives did not show significantly better awareness than healthy adults in these two aspects (p > .05). 

*Risk factors*

Among all participants, smoking, alcohol intake, and toxic environment were the three most recognised cancer risk factors (94.9%, 92.3%, and 89.7% respectively), while diabetes was the least (40%) (Table 4). Apart from that, physical exercise, good sleeping, and a balanced diet were the most commonly recognised factors that lower cancer risk (85.4%, 71.9%, and 83.1% respectively) while breed-feeding was the least (23.6%). 

Healthy individuals (18.46 ± 5.47)demonstrated higher awareness than cancer patients and relatives (16.37 ± 5.64) in recognizing risk factors associated with cancer, p< .001 (Table 4). More healthy individuals (59.2%) than cancerous patients and relatives (45.4%) understood that age is a risk factor linked with cancer, p = .002 (Table 4). More members from the general community (60.9%) than those related with cancers (51.8%) were aware of family history of cancer as a risk factor, p<.001. Papilloma virus, hepatitis, pancreatitis, and gastritis are other lesser-known risk factors of cancer among the cancer-related people as compared to the general community, p< .05 (Table 4). 

*General attitude towards cancer*

Many participants from both cancerous and healthy groups had positive attitude towards screening tests, with 73% disagreed that ‘Screening tests is unnecessary if having cervical vaccine’, 84.1% ‘Breast self-exam is the simplest method for breast cancer screening.’, and 74.7% ‘Cancer screening needs to be done for people > 50 years old.’ 

Noticeably, more healthy individuals (68.7%) than cancerous patients and relatives (58.2%) believed that a person can still continue working while undergoing treatment (Table 4). In contrast, much fewer healthy adults than cancer-related adults reported having been performed cancer screening test (15.5% vs. 26.9%, respectively, p<.001), having been provided with information on cancer diagnosis and treatment (43% vs. 51.8%, p = .046), and having trust on existing diagnosis measures (38% vs. 69.1%, p<.001). In term of cancer secondary prevention, although 88% of general population believed cancer screening tests being useful, but only one half of general population were confident in the diagnosis measurements provide by health care systems; and less of 20% of population have performed cancer screening test.

**Figure 1:**
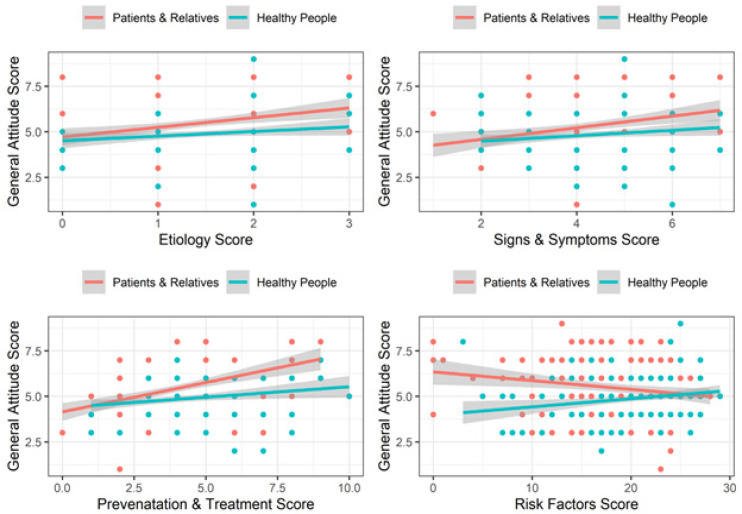
Association between General Attitude Score and Others

To evaluate which factors have association with general attitude towards cancer, the results from the linear regression model showed that knowledge about etiology, signs and symptoms, prevention and treatment and risk factors significantly associated with attitude of population, except among cancer patient and relatives group, knowledge about risk factor have negative association with attitude towards cancer (Figure 1).

**Table 1:** Sociodemographic Data of Cancer Patients, Relatives and Healthy People

Variables	No. (%) (N = 533)		P-value
	Patients& Relatives (n = 249)	Community (n = 284)	
Age (SD)	42.7(15.0)	34.4(13.4)	<0.001
Age group			<0.001
<30 years	62 (24.9)	135 (47.5)	
30-60 years	137 (55.0)	127 (44.7)	
>60 years	29 (11.6)	10 (3.5)	
Gender			
Female	177 (71.1)	173 (60.9)	
Male	67 (26.9)	110 (38.7)	
Educational level*			<0.001
None	9 (3.6)	0 (0)	
Primary	40 (16.1)	17 (6.0)	
Secondary	51 (20.5)	27 (9.5)	
Highschool	70 (28.1)	114 (40.1)	
College	26 (10.4)	24 (8.5)	
University	47 (18.9)	101 (35.6)	
Income (USD)			0.004
217 and below	83 (33.3)	64 (22.5)	
218 to 435	74 (29.7)	92 (32.0)	
436 to 870	19 (7.6)	45 (15.8)	
871 to 1304	9 (3.6)	6 (2.1)	
1305 and above	8 (3.2)	4 (1.4)	
Prefer not to say	56 (22.5)	74 (26.1)	
Employment status			<0.001
Full-time	65 (26.1)	120 (42.3)	
Part-time	19 (7.6)	30 (10.6)	
Housework	51 (20.5)	21 (7.4)	
Retired	21 (8.4)	14 (4.9)	
Unemployed	11 (4.4)	9 (3.2)	
Self-employed	42 (16.9)	19 (6.7)	
Others	30 (12.0)	66 (23.2)	
Marital status			<0.001
Single	62 (24.9)	147 (51.8)	
Married	160 (64.3)	123 (43.3)	
Separated	4 (1.6)	3 (1.1)	
Divorced	7 (2.8)	5 (1.8)	
Widow	7 (2.8)	3 (1.1)	
Others	9 (3.6)	3 (1.1)	

Notes: The differences between patients and relatives, and healthy individuals were assessed by multi nominal regression analyses.

**Table 2:** Knowledge Score between Patients and Relatives and Healthy Individuals

Knowledge score	Mean (SD)		p-value	Adjusted p-value*
	Patients & Relatives	Healthy People		
Etiology	1.39 (0.66)	1.39 (0.58)	0.636	0.971
Signs &symptoms	4.21 (1.25)	4.14 (1.17)	0.583	0.709
Prevention & treatment	3.87 (1.69)	3.95 (1.72)	0.656	0.363
Risk factors	17.9 (6.36)	19.9 (4.78)	0.001	0.005
General attitude	5.46 (1.43)	4.87 (1.26)	<0.001	<0.001
Total score	32.7 (6.69)	34.5 (5.27)	0.019	0.129

(*) P-value is adjusted for education level.

**Table 3:** Factors Influencing Performance of Total Knowledge Score among Cancer Patients Andrelatives and Healthy Groups

Factors	Mean of total knowledge score		Healthy people	p-value
	Patients &families	p-value		
Age group		0.639		0.073
<30 years	37		40	
30-60 years	36.5		37	
>60 years	35		43	
Gender		0.883		0.741
Female	37		39	
Male	36		39	
Educational level*		<0.001		<0.001
None				
Primary	30		34	
Secondary	34		31	
Highschool	36.5		40	
College	38		37	
University	39		41	
Income (USD)		<0.001		0.323
217 and below	34		41	
218 to 435	39		38	
436 to 870	38		39	
871 to 1304	45		41	
1305 and above	39		44.5	
Prefer not to say	33		37.5	
Employment status		0.015		0.66
Full-time	39.5		38	
Part-time	35.5		37.5	
Housework	34		37	
Retired	35.5		41	
Unemployed	33		43	
Self-employed	36		40	
Others	32		40	
Marital status		0.038		0.192
Single	37		40	
Married	36		38	
Separated	38		31	
Divorced	25		34	
Widow	25		41	
Others	37		37	

Notes: Kruskal–Wallis (K–W) test was used to assess the effect of age, educational level, employment status, income level, and marital status on the total knowledge score. Mann–Whitney (M–W) U-test was used for gender.

**Table 4:** Knowledge Distribution in Three Groups

Variables	Total (N = 533)	Patients & families (n = 249)	Healthy people (n = 284)	p-value
Etiology				
Cancer is largely due to genetic	196 (36.8)	86(34.5)	110 (38.7)	0.324
Cancer is due to uncontrolled cell growth	483 (90.6)	225 (90.4)	258 (90.8)	0.882
Cancer is an infectious disease	475 (89.1)	212 (85.1)	263 (92.6)	0.008
Signs and symptoms				
Always painful	298 (55.9)	122(49.0)	176 (62.0)	0.003
No breast cancer in men	357 (67)	170 (68.3)	187 (65.8)	0.580
Only women aged 50+ yr have breast cancer	439 (82.4)	198 (79.5)	241 (84.9)	0.112
Cancer is only diagnosed when symptoms are clear	270 (50.7)	107 (43.0)	163 (57.4)	0.001
Understand about pathology biopsy	413 (77.5)	184 (73.9)	229 (80.6)	0.077
Understand about stage of cancer	500 (93.8)	230 (92.4)	270 (95.1)	0.212
Understand about Pap smear for cervical cancer screening	348 (65.3)	149 (59.8)	199 (70.1)	0.014
Understand about fecal occult blood test for colorectal cancer screening	193 (36.2)	61 (24.5)	132 (46.5)	<0.001
Prevention and treatment				
Cancer is preventable	461 (86.5)	211 (84.7)	250 (88.0)	0.310
There have been vaccin for cervical cancer	414 (77.7)	183 (73.5)	231 (81.3)	0.037
Cancer is uncurable	445 (83.5)	208 (83.5)	237 (83.5)	1.000
Treatment will cause cancer to spread	471 (88.4)	217 (87.1)	254 (89.4)	0.420
Cancer can be curable by herb	371 (69.6)	180 (72.3)	191 (67.3)	0.221
Fasting to starve cancer cells	476 (89.3)	230 (92.4)	246 (86.6)	0.035
Surgery will cause cancer to spread	409 (76.7)	184 (73.9)	225 (79.2)	0.152
Cancer treatment must have radio- or chemotherapy	171 (32.1)	72 (28.9)	99 (34.9)	0.142
Non-surgical cancer treatment means not to be treated by other ways	408 (76.5)	181 (72.7)	227 (79.9)	0.052
Understand about palliative care	250 (46.9)	109 (43.8)	141 (49.6)	0.192
Risk factors				
Aging	281 (52.7)	113 (45.4)	168 (59.2)	0.002
Regular exercise	455 (85.4)	210 (84.3)	245 (86.3)	0.541
Smoking	506 (94.9)	228 (91.6)	278 (97.9)	0.001
Alcohol consumption	492 (92.3)	225 (90.4)	267 (94.0)	0.142
Overweight, obesity	351 (65.9)	155 (62.2)	196 (69.0)	0.120
Sleeping well	383 (71.9)	178 (71.5)	205 (72.2)	0.923
Balanced diet	443 (83.1)	206 (82.7)	237 (83.5)	0.908
Less vegetable diet	285 (53.5)	130 (52.2)	155 (54.6)	0.602
Salty diet	290 (54.4)	140 (56.2)	150 (52.8)	0.434
Eating a lot of red meat	280 (52.5)	132 (53)	148 (52.1)	0.862
Eating a lot of sea food	234 (43.9)	99 (39.8)	135 (47.5)	0.080
Stressful	308 (57.8)	134 (53.8)	174 (61.3)	0.095
Exposure to environment hazards	478 (89.7)	216 (86.7)	262 (92.3)	0.045
History of cancer	295 (55.3)	129 (51.8)	166 (58.5)	0.138
Family history of cancer	282 (52.9)	109 (51.8)	173 (60.9)	<0.001
Papilloma virus infection	218 (40.9)	90 (36.1)	128 (45.1)	0.042
Hepatitis	345 (64.7)	143 (57.4)	202 (71.1)	0.001
Pancreatitis	292 (54.8)	122 (49.0)	170 (59.9)	0.015
Gastritis	333 (62.5)	141 (56.6)	192 (67.6)	0.009
Diabetes	213 (40.0)	95 (38.2)	118 (41.5)	0.427
Plastic, disposable items	316 (59.3)	143 (57.4)	173 (60.9)	0.428
Hair dye	355 (66.6)	157 (63.1)	198 (69.7)	0.118
Mobiphone, wifi	142 (26.6)	63 (25.3)	79 (27.8)	0.556
Xray, radiation	354 (66.4)	143 (57.4)	211 (74.3)	<0.001
Multiple pregnancies	251 (47.1)	115 (46.2)	136 (47.9)	0.728
Breast feeding	126 (23.6)	44 (17.7)	82 (28.9)	0.003
Longterm OCP use	229 (43)	105 (42.2)	124 (43.7)	0.793
Multiple sexual partners	247 (46.3)	107 (43.0)	140 (49.3)	0.164
Organic foods	226 (42.4)	89 (35.7)	137 (48.2)	0.004
Transgenic foods	135 (25.3)	50 (20.1)	85 (29.9)	0.010
Functional foods	174 (32.6)	65 (26.1)	109 (38.4)	0.003
General attitude				
Undergoing treatment cannot continue working	340 (63.8)	145 (58.2)	195 (68.7)	0.015
Screening tests is unnecessary if having cervical vaccin	389 (73.0)	177 (71.1)	212 (74.6)	0.380
Breast self-exam is the simplest method for breast cancer screening	448 (84.1)	214 (85.9)	234 (82.4)	0.287
Cancer screening needs to be done for people > 50 years old	398 (74.7)	185 (74.3)	213 (75.0)	0.921
Cancer screening is useless	63 (11.8)	34 (13.7)	29 (10.2)	0.229
I have been done cancer screening test	111 (20.8)	67 (26.9)	44 (15.5)	0.001
I have got information on cancer diagnosis and treatment	251 (47.1)	129 (51.8)	122 (43.0)	0.046
I trust on existing diagnostic measures	280 (52.5)	172 (69.1)	108 (38.0)	<0.001

## Discussion

In the context of cancer being a major public heath challenge and the substantial gap between current evidence – based recommendations and public perceptive are further highlight by increasing awareness of basic knowledge of cancer etiology, risk factors, signs and symptoms, andgeneral attitude towards cancer, which is essential for primary prevention as well as early detection disease. The study was conducted to evaluate the awareness of these factors among the general community, cancer patients, and their relatives in Vietnam and found that although understanding of cancer is relatively good, a number of misconceptions and misunderstanding persist. Cancer patients were lower awareness of risk factors but more positive attitude towards cancer as compared to their healthy counterparts. The results highlight the need to improve public health educationin view of lack of awareness about the role of primary prevention and early detectionin the community.

In an exhaustive review of more than 500,000 scientific studies, the World Cancer Research Fund (WCRF) maintained that, despite of the multifactorial etiology of cancer, most cancers – between 65% and 70% - can be prevented by avoidance of tobacco in any form, non-excessive consumption of alcohol, combined with appropriate diet, physical activity, and body composition (Robb et al., 2009). Previous studies have proposed the link between poor public awareness of risk factors, prevention, screening, and general attitudes towards cancer to higher incidence of infection-related cancer (preventable), delayed presentation (presentation at more advanced stages of the disease), and worse outcomes (Robb et al., 2009). This is especially important in low and middle-income countries such as Vietnam where systematic cancer screening programs are not available and limited resources exist to support patients with cancer with advanced stages of cancer (Tran et al., 2020). 

Our study’s overall findings indicate that there was more awareness and knowledge among healthy adults compared to those living with cancer or having family members with cancer (cancerous group) even after being adjusted for educational level.In line with the existing literature (Blake et al., 2015; Linsell et al., 2008; Webster and Austoker, 2006), educational level is associated with level of cancer knowledge and awareness across two groups in this study. Cancerous adults and healthy adults did not differ in their knowledge of prevention and treatments, perhaps because of having direct experience with cancer, patients and their families might have been familiarised with cancer talks related to prevention and treatment methods. 

Most of respondents were aware that cancer is characterized by uncontrolled cell growth and that cancer is of non-contagious nature. This is probably a common knowledge because of the prevalence of cancer in Vietnam and its implication of cancer as a disease that has no specific causes or symptoms but a general condition of malfunctioning cells that could happen at any anatomical locations within the body. In contrast, much fewer people know that cancer has genetic origin. For one thing, it might be quite challenging to fully comprehend the conceptualisation of gene expression under certain environmental exposures. For another thing, although a correlation between genetic factors and cancer onset is established in various studies, cancer molecular biology research remains limited in Vietnam (Tran et al., 2020). However, the current literacy showed that besides external environmental factors, the internal genetic has an important role leading to cancer. About half of all cancers are due to unknown risk factors or carcinogen (such as physical, chemical of infectiuos agents) but due to spontaneous mutations during cell division (Tomasetti et al., 2017). Understanding half of cancers can not be prevented, we have to accept that primary prevention strategies can not be isolated from an overall approach that including access to screening, to identify cancer early. More importantly, it means everyone needs to be vaccinated and screening for cancer when possible even if they are at low risk because of family history and their own healthy habits. This lack of understanding can contribute partly to explain why less of 20% of population have performed cancer screening test.

Overall, the vast majority of respondents know about the stages of cancer, pathology biopsy, and breast cancer risk across ages. Perhaps such knowledge is partly attributed to a few communicative screening and awareness programs for the general community during the past 10 years (Pham et al., 2019). That said, more concerted and long-term efforts that bring in experts, non-state sectors, and the government are required if cancer awareness is to be significantly improved. The perception that cancer can only be diagnosed with clear signs or that cancer is always associated with pain might prevent patients from having their health checked when the slightest symptoms first appear. A retrospective study on cancer stages in Vietnam reported that most cancer patients present themselves at stages III and IV of cancer (Bui et al., 2015). Respondents had general knowledge (‘cancer is curable’, ‘cancer is preventable’) of cancer prevention and treatment, yet they were poor at the specific details (‘Cancer treatment must have radio- or chemotherapy’, ‘The goal of palliative care is to cure cancer’). Importantly, patients and families generally did not have better knowledge of cancer treatment compared to their healthy counterparts. The lack of awareness in other methods of treatment besides chemotherapy and radiation therapy might reflect the lack of expertise availability of modern techniques for immune histochemistry and molecular analysis at many provincial care centers (Pham et al., 2019). These findings can be useful in promoting future community dialogues about screening, vaccination, and early presentation in the future; meanwhile, they are suggestive of further investment for cancer treatment in Vietnam. 

Our findings of low level of awareness of cancer risk factors among the general population, healthy and cancerous alike, concur with previous research (Elshami et al., 2020; Ravichandran et al., 2011; Richards et al., 2017). Except for very well-known contributors that have been campaigned worldwide during the last few decades: nicotine intake, alcohol consumption, and toxic environment, only a limited number of participants in bothgroups were able to identify modifiable risk factors of cancer diseases. Educational intervention programs have been credited for the reduced incidence of infection-related cancers such as of the lung, cervix, liver, and stomach in countries with high HDI (WHO, 2020). However, people seem not to be aware of eating habits as contributing to cancer risk which also contributes to worsecancerous outcomes. Diabetes were not recognized as a risk factor by more than half of respondents. There were still many misconceptions, up to 35% of population did not know obesity being an important risk factor of cancer; one third identified ‘cancer can be cured by herb’ and 23% believed ‘surgery will cause cancer to spread’.Poorawareness and knowledge of risk factors related to cancer have been reported elsewhere in both developed and developing countries among healthy people and healthcare providers (Blake et al., 2015; Wardle et al., 2001). The current finding that healthy and cancerous people have limited awareness of both non-modifiable (gene, age) and modifiable factors has two important implications. 

Generally, people with cancer experiences displayed more positive attitude towards cancer as compared with their healthy counterparts. More patients and their relatives than the general community said they did screening, probably because of or after their family members’ cancer incidence. More cancerous people than healthy people expressed positive attitudes towards existing cancer diagnosis methods. However, more patients and relatives than healthy people thought that undergoing cancer treatment equals to having an inability to continue working. Perhaps with most cancer cases being diagnosed at advanced stages, patients often find themselves unable to continue working due to severity of the disease as well as practical issues such as having to leave their hometowns for treatment in specialised hospitals. It is interesting that there was the negative association between attitude towards cancer and awareness of risk factors in patients and relatives. It may be explained partly when the patients had knowledge about cancer and healthy lifestyle, they would get frustrating and even angry to face cancer. The changes in emotion and mood made them having negative attitude towards cancer. Once again, this highlights the need to improve public awreness about etiology of cancer; everyone can be at risk of cancer, disease can caused by gene faults that develope during the time because of random errors in cell replication and no because of bad lifestyle, diet chemicals or inherited genes; and secondary prevention is important.

The diversity of knowledge content covered in this survey is both a strength and a weakness of our study. It presents one of the first studies about this topic in Vietnam from where future research can explore. Even though individual’s awareness and knowledge alone might not be sufficient on its own to establish preventative behaviours and to reduce cancer incidence, they critically contribute to the success of other more systematic health promotion strategies from other sectors.

In conclusion, this study provided some preliminary information about the current level of public awareness of cancer in Vietnam. Knowledge about cancer and its risk factors should be improved among the general population as well as among those with direct experiences with cancer. The findings provide useful information about the public perception of cancer and serve as a starting point to better understanding the public perspective.
